# Supplementary material for: Gut microbiota and derived metabolites mediate obstructive sleep apnea induced atherosclerosis
Source: Gut Microbes. 2025 Mar 2;17(1):2474142. doi: 10.1080/19490976.2025.2474142 (PMC11881840; doi:10.1080/19490976.2025.2474142)
Supplement: Supplemental Material [file KGMI_A_2474142_SM0621.zip › Supplementary_Figures_and_Legends_Revision_ clean.docx]

Supplementary Figures and Legends

**
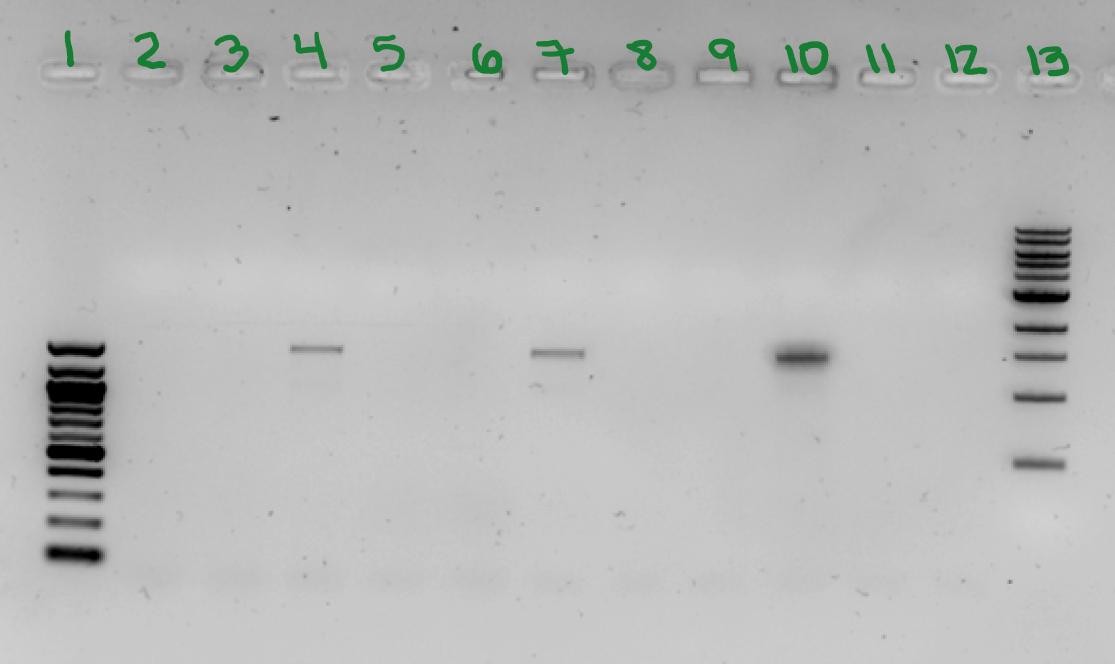
**

**Supplementary Figure S1. Sterility control of germ-free mice.** To monitor germ-free status, fecal samples were collected bi-weekly and tested by 16S PCR using Zymo Research Quick-DNA Fecal/Soil Microbe Miniprep Kit (D6010, Zymo Research, Irvine, CA) according to manufacturer’s instructions. The primer sequences are 27F: 5’-AGAGTTTGATCMTGGCTCAG- 3’ and 1492R: 5’-GGTTACCTTGTTACGACTT-3’ (1:1000 dilution). Less than 50 mg of fecal samples (about half of a pellet) were used. Above is a representative gel picture of 16S PCR. Line 1, 100 bp ladder; Lines 2-9, fecal samples from different germ-free cages at different time points. Lines 4 and 7 showed the bands, suggesting bacterial contamination, these samples were from the same cage at week 2 and week 4 treatment, we removed these mice from the experiment and replaced another set of germ-free mice; Line 10, positive control (fecal sample from conventionally-reared cage); Line 11, negative control (fecal sample from germ-free cage); Line 12, blank and Line 12, 1 KB ladder.


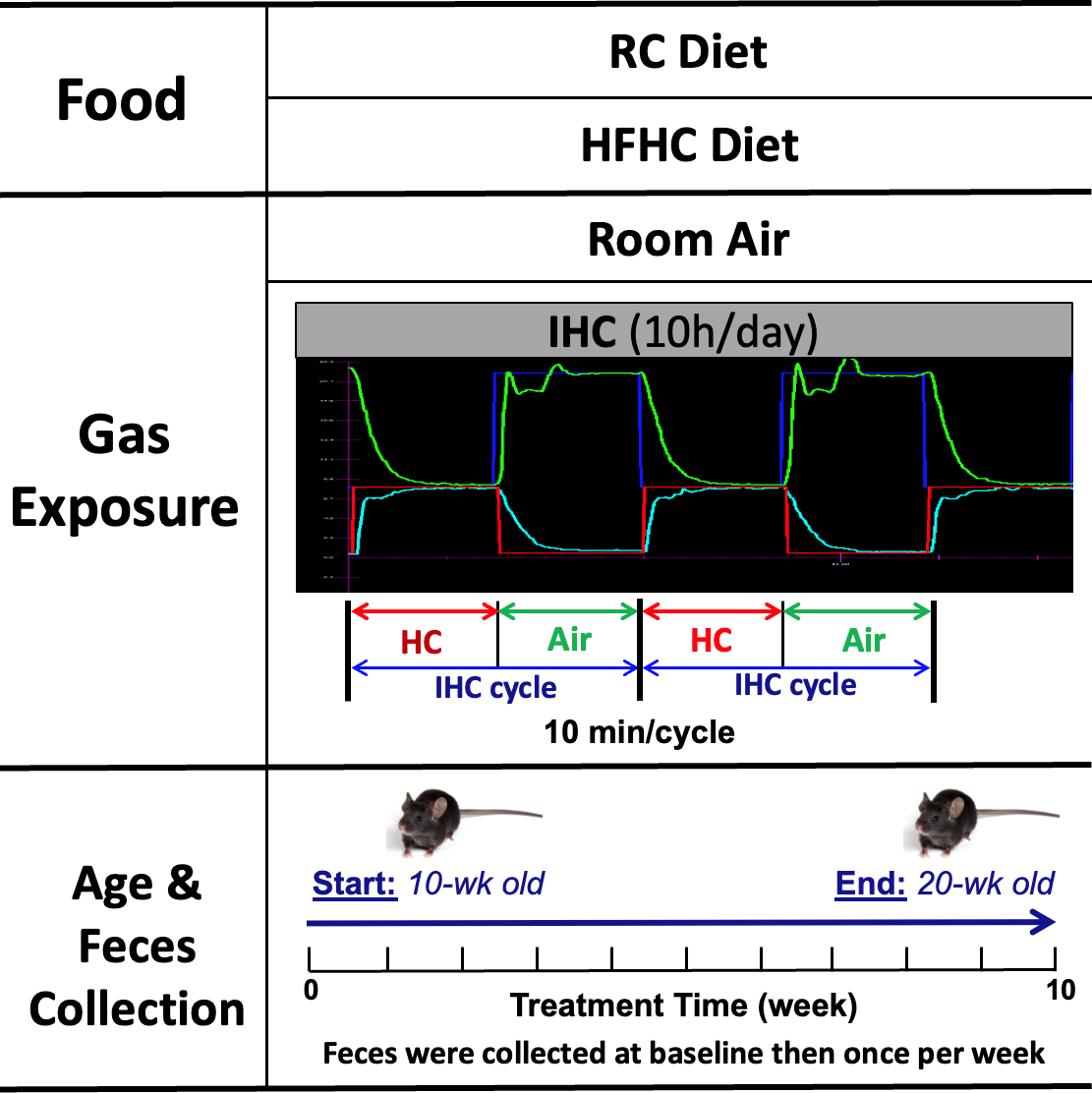


**Supplementary Figure S2. Schematic illustration of treatment paradigm.** At 10 weeks of age, mice were treated with high fat high cholesterol diet (HFHC) with or without intermittent hypoxia and hypercapnia (IHC). The treatment period lasted for 10 weeks. IHC exposure was administrated as short periods (∼4 minutes) of 8% O2 and 8% CO2 separated by alternating periods (∼4 minutes) of normoxia (21% O2) and normocapnia (0.5% CO2) with 1–2 minutes ramp intervals, 10 minutes per cycle, 10 hours per day during the light cycle (The blue line was the O2 set point and the green was the actual level of O2. The red line was the CO2 set point and light blue was the actual level of CO2). The control groups remained in room air either on HFHC diet or regular chow (RC) for the same period. At the end of the experiment (20 weeks of age), the mice were perfused and the blood vessels were dissected out, stained and analyzed for atherosclerotic lesions. Fecal samples were collected at the baseline then once per week and used for microbiome and metabolomics analyses.


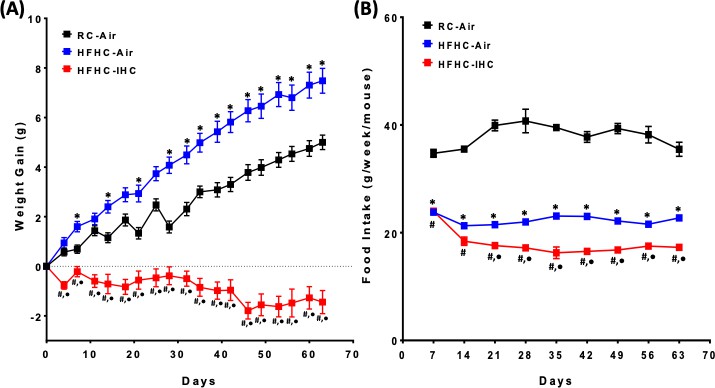


**Supplementary Figure S3. (A) Body weight gain and (B) Food consumption.** RC-Air vs HFHC-Air vs HFHC-IHC. Mice on the HFHC diet showed greater weight gain than those in the RC group. IHC with HFHC caused weight loss over the treatment time. More RC food was consumed than HFHC food. Under the same HFHC condition, food intake was reduced by IHC exposure starting week 3 of the treatment. Multiple t-tests with Bonferroni correction, P<0.05, * HFHC-Air vs RC-Air, # HFHC-IHC vs RC-Air and **·** HFHC-IHC vs HFHC-Air.


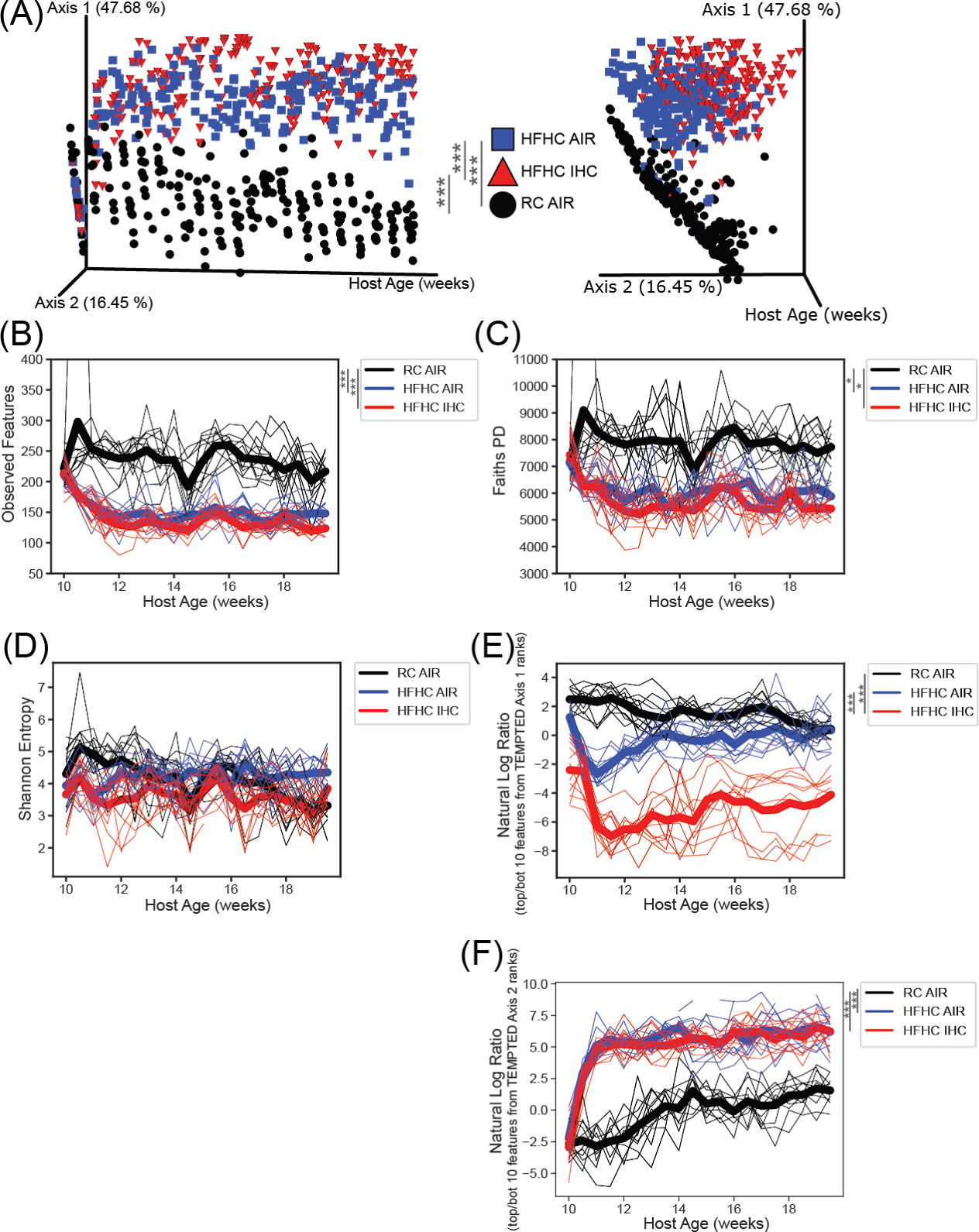


**Supplementary Figure S4. Additional 16S V4 microbiome analysis.** (A) Weighted UniFrac Beta Diversity PCoA metric takes into account abundance and phylogeny. Each dot represents a single sample. Lateral view on left, end-on view on right. PERMANOVA was used to determine significance. Alpha Diversity Metrics: (B) Observed Features (unique ASVs) (C) Faith’s

Phylogenetic Diversity, and (D) Shannon Entropy. The thick line represents the mean of all mice in the group and the thin lines represent individual mice over time. Linear mixed effect model (equation: alpha diversity metric value ~ host_age * diet_exp + (1|host_subject_id)) was used to determine significant differences across time. (E) Natural Log Ratio of the top and bottom 10 differentially ranked ASVs as present in TEMPTED Axis 1. (F) Natural Log Ratio of the top and bottom 10 differentially ranked ASVs as present in TEMPTED Axis 2. See Supplementary Tables S1 and S2 for ASVs and taxonomic annotation. The thick line represents the mean of all mice in the group and the thin lines represent individual mice over time. Linear mixed effect model (log_ratio ~ host_age * diet_exp + (1|host_subject_id)) was used to determine significant differences across time. Significance: ns p > 0.05; * p < 0.05; ** p < 0.01; *** p < 0.001.


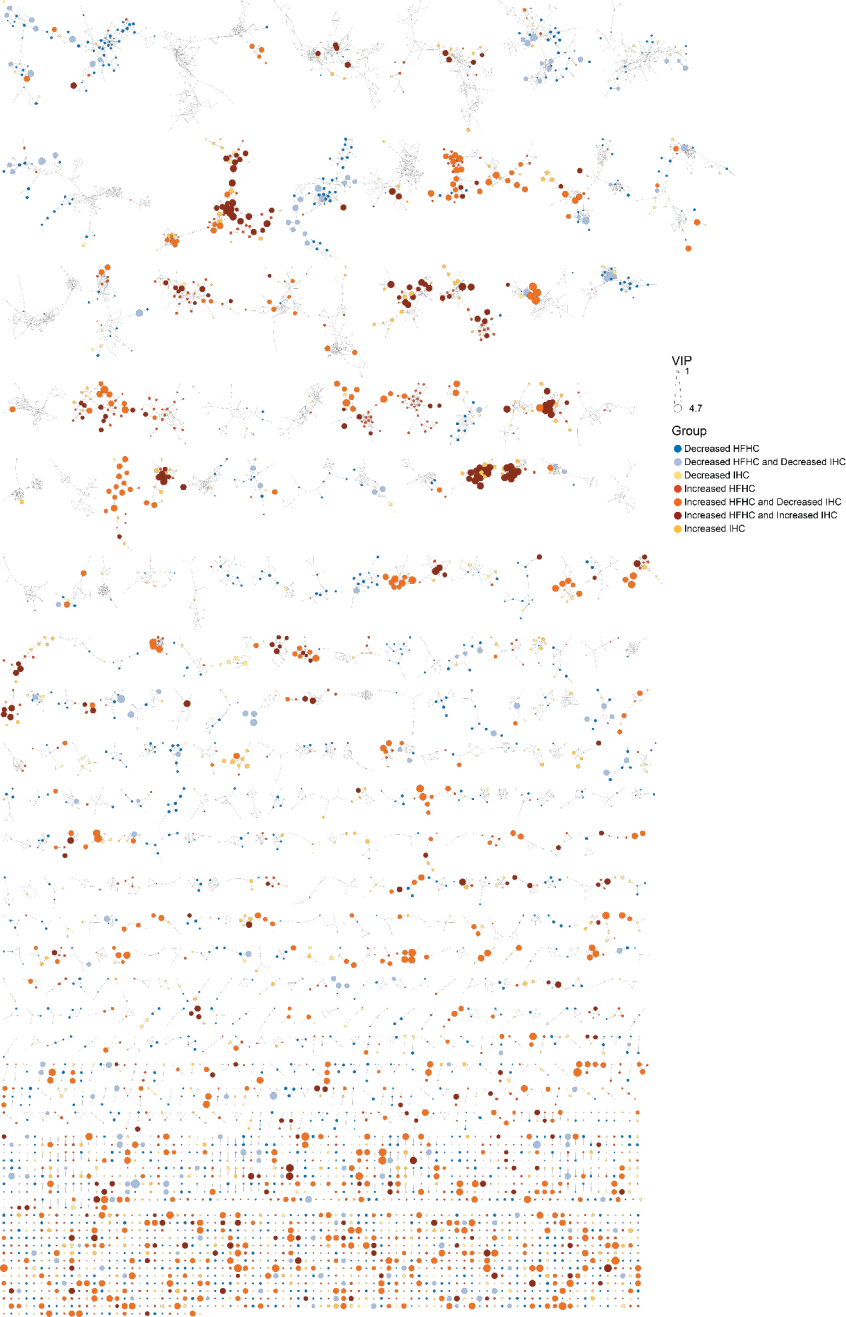


**Supplementary Figure S5**. **Molecular network.** Molecular network filtered for significant features from the generated PLS-DA models. Variable importance represented by size of nodes (VIPs [1,4.7]). Colors indicate groups in which the features were present in higher abundances. A full explorable and interactive Cytoscape file is available to download on GitHub (<https://github.com/simonezuffa/Manuscript_HFHC_IHC>).


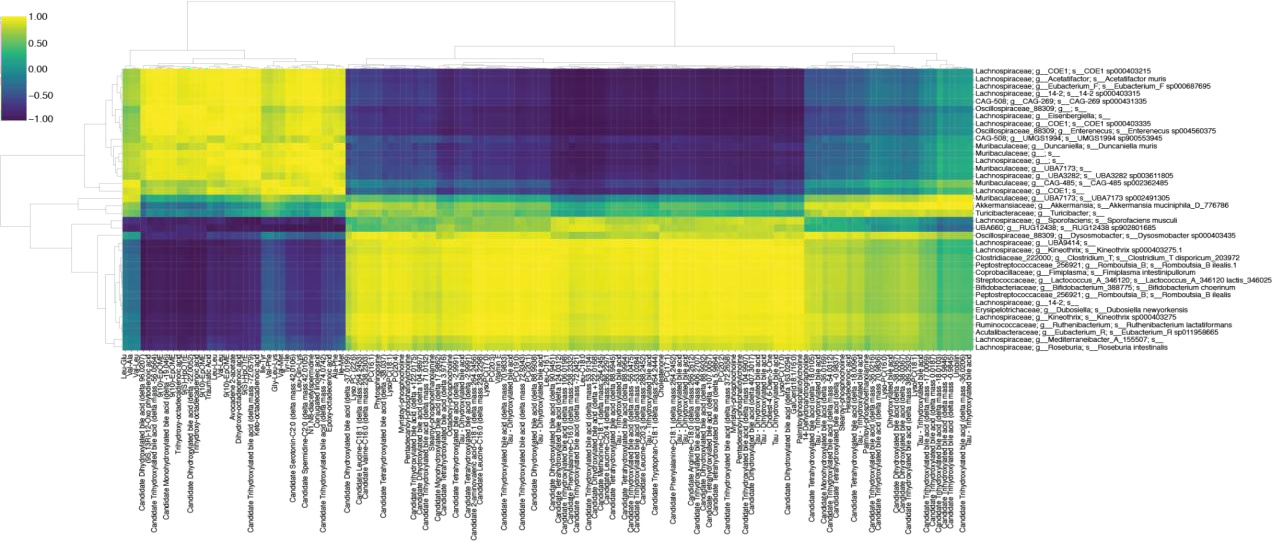


**Supplementary Figure S6. Joint-RPCA of top microbes and metabolites of interest.** All ASVs of interest (y-axis) and used in any of the log ratios (x-axis; Figure 2E-F, S2E-F) and their co- variance with all metabolites of interest (Figure 3C, Supplemental Table 1-2) based on the data from the final time point in common (host age = 19.5 weeks) using joint-RPCA.


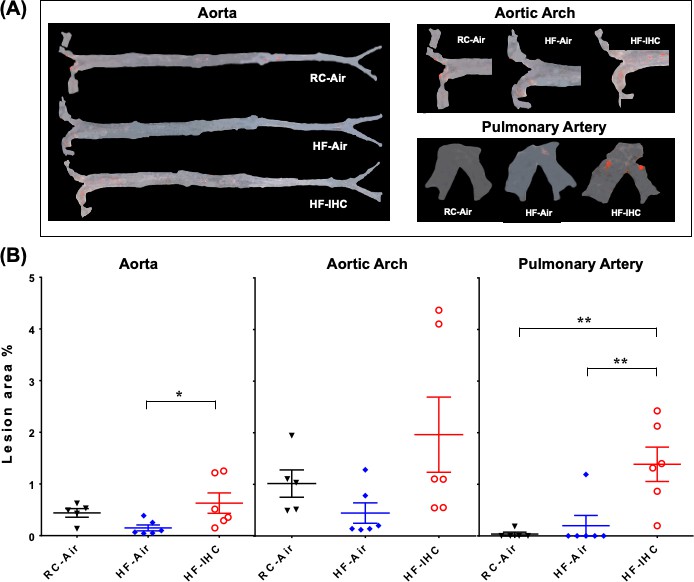


**Supplementary Figure S7. Atherosclerotic lesions after 10-week of high fat (HF) diet with or without IHC in SPF *ApoE*-/- mice.** (A) Representative Sudan IV-stained images of lesions. (B) The en-face lesions were quantified as the percentage of lesion area in the total area of the blood vessel examined. The HF diet caused less than 5% lesion formation. IHC promoted atherosclerotic progression in the presence of HF compared to the controls in the aorta and pulmonary artery. Data are presented as means ± SEM. Statistical significance tested via One-way ANOVA followed by Tukey’s multiple comparison test. Significance: *p<0.05, ** p < 0.01.
